# Cardiac Stereotactic Body Radiation Therapy (SBRT) for Refractory Ventricular Tachycardia Using Innovative Motion Tracking With Electroanatomical Mapping and CyberKnife Synchrony

**DOI:** 10.7759/cureus.87275

**Published:** 2025-07-04

**Authors:** Chaewon Hwang, Gleb A Kuzmin, Timothy Maher, Nima Aghdam

**Affiliations:** 1 Radiation Oncology, Tufts Medical Center, Boston, USA; 2 Radiation Oncology, Beth Israel Deaconess Medical Center, Harvard Medical School, Boston, USA; 3 Cardiology, Beth Israel Deaconess Medical Center, Harvard Medical School, Boston, USA; 4 Radiation Oncology, NYCyberknife at New York University Perlmutter Cancer Center, New York City, USA

**Keywords:** cardiac sbrt, cardiorespiratory motion tracking, cyberknife® radiosurgery, cyberknife synchrony, electroanatomical mapping, sbrt (stereotactic body radiotherapy), ventricular tachycardia (vt)

## Abstract

Ventricular tachycardia (VT) is a potentially fatal arrhythmia that often requires a combination of medications and invasive procedures, including implantable cardioverter defibrillators (ICDs) and radiofrequency catheter ablation, to manage and treat. Despite these interventions, recurrence rates for VT remain high, particularly in patients with structural heart disease. Recent advancements in cardiac stereotactic body radiation therapy (SBRT) offer a promising alternative for patients with refractory VT, particularly for those whose arrhythmogenic areas are difficult to access through conventional methods. This study presents a novel approach to treating refractory VT, integrating advanced cardiorespiratory motion tracking using CyberKnife Synchrony (Accuray Incorporated, Madison, WI, USA) and a 3D heart reconstruction technology (inHeart, Pessac, France). The case involves a 66-year-old male with a history of dilated cardiomyopathy and recurrent VT, unresponsive to multiple medical and invasive interventions, including ICD implantation and catheter ablation. Using a combination of CT imaging, MRI, and electroanatomical mapping, we identified the arrhythmogenic region of the heart. He then underwent a single fraction of SBRT, 25 Gy to the target area, using real-time motion tracking via Synchrony. Six months post-treatment, the patient remained free from VT and has not required ICD therapies or further medical or procedural interventions. Our center has successfully implemented cardiac SBRT for refractory VT using inHeart technology for heart reconstruction and Synchrony for cardiorespiratory motion tracking.

## Introduction

Ventricular tachycardia (VT) is a life-threatening arrhythmia with an unmet need for effective treatments. The combination of medications with invasive interventions such as catheter ablation is used to treat VT, targeting arrhythmogenic areas of the heart to eliminate re-entrant circuits. While implantable cardioverter defibrillators (ICDs) can terminate VT and radiofrequency catheter ablation helps reduce ICD shocks, long-term recurrence rates remain above 50% in patients with structural heart disease, as shown in multiple trials [1-5]. Additionally, such interventions can add significant morbidity to the patient, as these are invasive procedures.

Cardiac stereotactic body radiotherapy (SBRT) is an innovative treatment that uses non-invasive, high-dose radiation to treat the transmural area of the heart, targeting a specific area of the VT circuit, potentially treating areas that are too extensive or difficult to access through catheter ablation [6-13]. However, due to its newer approach, its use has not yet been standardized, and differences exist in tracking cardiorespiratory motion to deliver a safe dose of radiation, as well as in target delineation of the arrhythmogenic area of the heart [14]. For example, a systematic review and meta-analysis of prospective trials conducted by the STOPSTORM.eu consortium revealed a high level of heterogeneity and variation in target volume delineation methods, integration of electrophysiology data, and radiation delivery, despite the high efficacy of the treatment [14].

In this study, we used a novel approach of cardiorespiratory motion tracking to treat VT, using both inHeart technology (inHeart, Pessac, France) to provide a 3D reconstruction of the heart registered to prior electroanatomic mapping to identify and segment the arrhythmogenic area of the heart, and Synchrony, which is a technology built into CyberKnife (CK) (Accuray Incorporated, Madison, WI, USA), to track the motion of the heart during the respiratory cycle. More specifically, Synchrony utilizes imaging and artificial intelligence to track and adjust the treatment beam to the target's position, ensuring the accurate delivery of radiation, even during patient motion. In brief, inHeart was used to create an AI-assisted model of the patient's heart from ECG-gated cardiac CT and cardiac MRI, which was then fused with the planning CT in Precision, a planning software for CK, to guide target delineation. Synchrony was used to track cardiorespiratory motion in real-time, using the patient's ICD lead as a "fiducial marker" to safely treat refractory VT with cardiac SBRT using CK.

## Case presentation

The patient is a 66-year-old male with a history of dilated cardiomyopathy diagnosed in 2010 after experiencing exertional light-headedness while training for the Boston Marathon. At the time, a stress test showed premature ventricular contractions, and an echocardiogram revealed a reduced left ventricular ejection fraction of 35-40%. The normal left ventricular ejection fraction is ≥50%, per the American College of Cardiology, American Heart Association, and Heart Failure Society of America [15]. He reported no preceding episodes of chest pain, dyspnea, nausea, or vomiting, or any other protracted symptoms potentially associated with a myocardial infarction, nor any preceding severe viral infections. He followed up with his cardiologist at Lowell General Hospital after his diagnosis of dilated cardiomyopathy in 2010 and only had occasional palpitations, for which he wore two to three Holter monitors at various times, which showed a low burden of premature ventricular contractions. No cardiac treatment was initiated.

He continued experiencing worsening light-headedness, with a significant episode in 2020 during a bike ride, where he was found hypotensive with monomorphic VT (200-220 bpm), which was successfully externally cardioverted. Cardiac catheterization showed only mild coronary artery disease. Electrophysiology mapping failed to reproduce the VT, and a limited endocardial substrate ablation was performed; cardiac biopsies revealed nonspecific fibrosis. He had a single-chamber ICD implanted for secondary prevention and was started on metoprolol, sacubitril/valsartan, and atorvastatin. He remained VT-free until January 2024, when he had a symptomatic episode during marathon training. VT then recurred in March of 2024, and he underwent a redo epicardial ablation. More episodes of VT occurred in August and September of 2024, requiring repeat endocardial VT ablation and adjunctive coronary venous ethanol injection near the VT circuit isthmus. However, he had a recurrence less than one week later, prompting the initiation of dofetilide and later mexiletine. Notably, more recent VT episodes in December of 2024 occurred at rest, raising concern, as he had previously experienced VT only with exertion. A 2024 PET scan was negative for active sarcoid, and genetic testing was also negative, effectively ruling out other potential causes of VT or nonischemic cardiomyopathy.

Despite multiple medical and invasive procedural interventions to treat VT, his episodes of VT were worsening, occurring even at rest, towards the end of 2024. At that point, the patient presented to the department of radiation oncology to be considered for cardiac SBRT for refractory VT.

Cardiac SBRT simulation and delineation

The patient was CT-simulated with and without contrast. A test on the CK was scheduled for two hours after completion of the CT scan, allowing time for the creation of a tracking verification test. For the test, the ends of the right ventricular septal ICD lead were identified and marked as “fiducials” in Precision for tracking. The patient was then positioned on the CK as they would be for treatment, with an initial setup using spinal anatomy (XSight Setup) followed by a shift to the target. A Synchrony respiratory model was built using the marked ICD to confirm that tracking was consistent and reproducible. Following the tracking verification test, the target was contoured.

For target delineation, the following were used to define the treatment volumes: CTebh-ivc, which was IV contrast at expiration CT for electroanatomical mapping (EAM), which is a process that creates a 3D virtual model of the heart, allowing for the mapping of electrical activity and anatomical structures; CTibh, which was the natural inspiration CT to sample cardiac and respiratory motion to aid in tracking the movement of the arrhythmogenic area of the heart, which would then be used in reference with Synchrony during treatment; and CTfb1-3: 3x native free-breathing CT to sample cardiac and respiratory motion. The following images were imported and fused in Precision software: EAM using inHeart technology, as well as cardiac MR and CT images. Preliminary electrical gross tumor volume, the arrhythmogenic area of the heart, which was the target, was contoured on registered EAM+cardiac MR/CT. The inHeart software was used, which utilizes the electrophysiology of the heart to create an AI-generated image of the heart, helping to identify arrhythmogenic areas. Deformable registration to contrast-enhanced expiration CT scan was carried out in Velocity software. The images were initially manually registered according to the ICD to be used for tracking using a rigid transformation. Following this, deformable image registration (DIR) was performed within a region of interest defined by a 1-cm margin around the heart. The employed DIR utilized a modified B-spline deformable algorithm with a mutual information metric for evaluating the similarity between registered images.

The final ITV, defined as the maximum detected difference from all four-motion assessment CT image series in six major anatomical directions (anterior, posterior, right, left, superior, and inferior), was used to expand the original CTV. For the final PTV, an additional isotropic margin of 2 mm was added to compensate for the following variables of uncertainty: LED signal-marker position correlation uncertainty, intra- and inter-observer variabilities, which are the major sources of uncertainty in the GDM method, and technological uncertainty.

Prescription and planning

**Figure 1:**
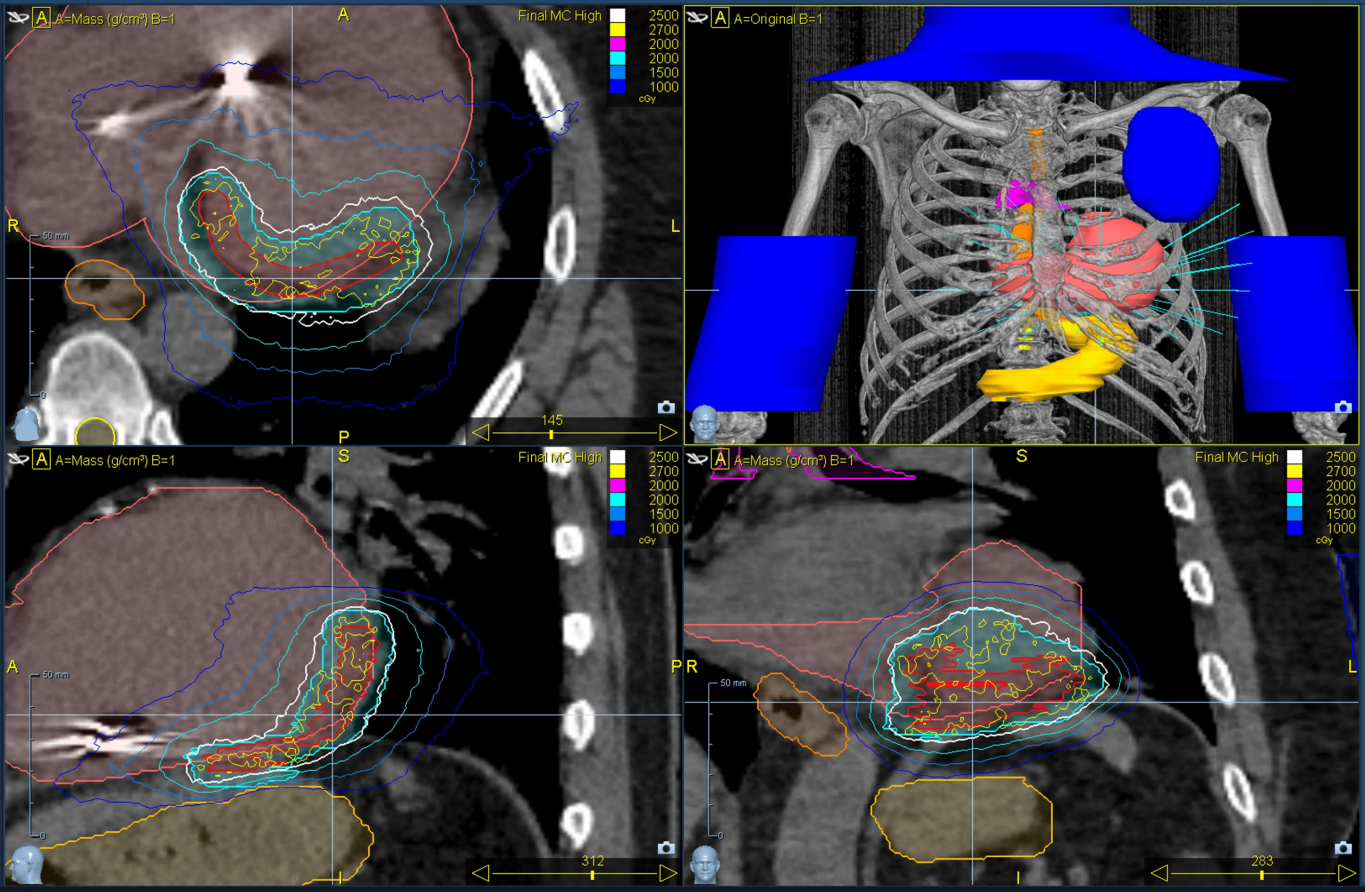
Cardiac SBRT target delineation and dose distribution The top left image shows an axial CT view of the heart, with the ICD located in the right ventricle and the arrhythmogenic area of the ventricle contoured, along with the dose distribution. The top right image is a 3D rendering that shows bony anatomy, including the organs at risk and the heart, contoured; the blue parts represent the contours used to block the entrance of the beam. Bottom left is a coronal view of the target contour, and bottom right is a sagittal view of the target contour, with dose distribution. SBRT: stereotactic body radiation therapy, CT: computed tomography, 3D: three dimensional

The prescription isodose line was 85% of the maximum dose, and the goal of treatment was to deliver at least 95% of the PTV a dose of 25 Gy in a single fraction. The planning was performed using Precision’s Monte Carlo dose calculation algorithm with CK’s InCise MLC collimator, resulting in 95.3% coverage of the PTV with the prescribed dose. SBRT target goals and dose constraints are shown in Table 1, and the cardiac target contour is shown in Figure 1. RTOG 0915 single fraction 34 Gy lung SBRT dose constraint was used as a reference, using conservative dose limits when appropriate to protect nearby organs at risk [16].

**Table 1:** Target goals and dose constraints for cardiac SBRT Dose constraints and target goals for cardiac SBRT. PTV was the target for cardiac SBRT, the arrhythmogenic area of the heart. The dose constraint and target goals were met as shown above. The value column refers to the goal constraint volume or dose that was achieved. For organs at risk other than the lung, the volume required to achieve the dose constraint goal is specified. For the lung, the constraints were a dose less than 700 cGy and 740 cGy, with critical volumes of 1500 cc and 1000 cc, respectively. SBRT: stereotactic body radiation therapy, PTV: planning target volume

Targets	Dose (cGy)	Dose (% of 2941 cGy max dose)	Volume (cm^3^)	Volume (%)	Dose constraint/target goal	Value	Goal met? (yes or no)
PTV	2500	85.0	67.03	95.3	Volume (%) ≥95.0	95.3%	Yes
Spinal canal	623	21.2	0.03	0.1	Dose (cGy) <1400	623 cGy	Yes
Esophagus	789	26.8	0.03	0.1	Dose (cGy) <1540	789 cGy	Yes
Trachea	76	2.6	4.00	22.3	Dose (cGy) <1050	76 cGy	Yes
Ribs	1030	35.0	1.00	0.9	Dose (cGy) <2200	1030 cGy	Yes
Skin	535	18.2	10.00	0.3	Dose (cGy) <2300	535 cGy	Yes
Lung receiving <700 cGy	5	0.2	2397.45	100.0	Volume (cm^3^) >1500.00	2397.45 cc	Yes
Lung receiving <740 cGy	5	0.2	2410.49	100.0	Volume (cm^3^) >1000.00	2410.49 cc	Yes
Stomach	1195	40.6	0.03	0.0	Dose (cGy) <1240	1195 cGy	Yes
Stomach	572	19.4	10.00	4.2	Dose (cGy) <1120	572 cGy	Yes

Motion tracking on the day of treatment

On the day of treatment, the patient was aligned using an XSight Setup and shifted to the Align Center of CK treatment. The end of the right ventricular septal ICD lead, identified in the initial tracking test, was used as the surrogate marker for tracking. A Synchrony respiratory motion model was constructed, establishing the correlation between the movement of external abdominal markers, which are three LED-based markers placed on the patient’s abdomen. A camera from CK tracks their motion to consider respiratory motion. Simultaneously, the position of the ICD lead within the heart is also tracked with X-rays that are taken. Then, a Synchrony model is built that correlates the LED-marker-based breathing motion with the fiducial-based target motion of the heart. The model was continuously updated throughout treatment, and both the patient's respiratory and cardiac function, as well as vital signs, were monitored via a closed-circuit monitor.

## Discussion

The patient was successfully treated in December 2024 with a single fraction of SBRT to the heart. Three months following the procedure, the patient has experienced no further episodes of VT and has not required ICD firing. During a recent follow-up with the cardiologist, six months post-treatment, the patient remained euvolemic and well-perfused, without further ICD firing. He had begun gradually increasing his exercise routine.

Since the publication of a successful cardiac SBRT for refractory VT [6,9], there have been further investigations into cardiac SBRT, analyzing different modalities of treatment delivery and VT characterization within the heart. In a review that listed standard linear accelerators (Linac), MR-Linac, and CK as treatment modalities of refractory VT, the pros and cons of such treatments were described. Regarding CK, it was mentioned that requiring invasive fiducial placement is a disadvantage [17]. However, we were able to circumvent this issue by utilizing the already existing ICD in the patient's heart as a fiducial marker for tracking using Synchrony.

According to a recent prospective cohort study, the estimated 10-year all-cause mortality following VT ablation is 39.4%. Mortality after VT ablation is worse in patients with structural heart disease and highest for patients with ischemic heart disease [18]. In a separate study that investigated the rate of recurrence of VT and predictors of VT recurrence, the rate of VT recurrence was 41.4% within a follow-up period of slightly more than three years. This study also found that persistence of late potentials (67% vs. 19%; hazard ratio 3.18 (2.18-6.65); P < 0.001) and lower left ventricular ejection fraction (30% (25%-40%) vs. 39% (30%-50%); P = 0.022) were predictive of VT recurrence [19]. These studies show a high all-cause mortality rate following VT ablation and a high rate of recurrence following VT ablation, which highlights the importance of investigating novel approaches for the treatment of VT.

Data on cardiac SBRT for refractory VT are still emerging, with no direct comparisons yet to outcomes from cardiac ablation. Currently, SBRT is considered only after failure of medical therapy and ablation. A systematic review of 157 patients treated with SBRT reported a one-year mortality rate of 32% (95% CI: 23-41), with nearly half of deaths occurring within three months; worsening heart failure was the leading cause (52%), and non-cardiac mortality was also notable (41%). Older age (≥70) was significantly associated with higher 12-month mortality (P < 0.022), while target volume and radiotherapy device had no significant impact [20]. Although these results cannot be directly compared to long-term cardiac ablation studies, they suggest SBRT may offer a viable treatment option for refractory VT, acknowledging that outcomes may be poorer given the advanced disease state of this population.

Additionally, a systematic review and meta-analysis on prospective trials has shown that although there is consensus among current prospective trials and case series regarding the safety and efficacy of cardiac SBRT, there is variability in the way outcomes are reported and defined, rendering comparisons among cardiac SBRT studies challenging [14]. As this approach to treating refractory cardiac VT is becoming more popular, there has been a need for a standardized guideline for cardiac SBRT. Recently, there has been a publication of a cardiac SBRT consensus statement discussing expert guidance on delineation of targets, patient selection, and acute and long-term monitoring of patient outcomes for those who underwent cardiac SBRT for refractory VT. It also provides a "table of advice" for cardiac SBRT [21]. Although the consensus guideline offers a wealth of evidence and explanation, the recommendations themselves leave room for interpretation, which is crucial for making the best clinical judgment regarding efficacy and safety. Our case has followed the guidelines set forth within the "table of advice." As with guidelines, as more data emerge, modifications and improvements to cardiac SBRT will be made. And although the STOPSTORM.eu consortium study has provided a helpful framework for evaluating cardiorespiratory motion during stereotactic arrhythmia radioablation [22], it was done on a phantom and therefore needs to be taken into clinical context as done with the prospective trials, case series, and review [9-14,17-20,23].

As with cardiac ablation with interventional cardiology, it is possible to have recurrence of VT following cardiac SBRT [14]. Within a Swiss cohort of twenty patients who had undergone cardiac SBRT for refractory VT, after a median follow-up of 25 months, 60% experienced a recurrence [23]. However, all of the recurrences were reported to be outside of the PTV. Therefore, it will be necessary to monitor for VT recurrence in our patient, keeping in mind the likely possibility of recurrence outside the area treated with cardiac SBRT. Although the mechanism of action for cardiac SBRT is unclear, one proposed mechanism is functional remodeling induced by cardiac SBRT that leads to improved conduction velocity [24]. However, more work needs to be done regarding the investigation of the mechanism of action of cardiac SBRT, which will allow us to better apply cardiac SBRT for treating refractory VT.

Of note, there are side effects such as pneumonitis and pericarditis reported secondary to cardiac SBRT [11]. In a prospective Phase I/II study that investigated the use of cardiac SBRT for treatment-refractory VT that enrolled 19 patients, two patients developed delayed grade 2 pneumonitis that resolved with steroids, and five patients developed pericardial effusion. Our patient, who underwent treatment, has not experienced any side effects since completing cardiac SBRT. However, longer follow-up is warranted to monitor for potential side effects, such as pneumonitis, pericarditis, and pericardial effusion.

This case demonstrates an innovative use of inHeart technology to digitally reconstruct the heart and integrate it with CK's Precision treatment planning software for accurate target delineation. To address the challenge of cardiorespiratory motion during SBRT, the Synchrony motion-tracking system, typically used for respiratory and abdominal tracking, was adapted to follow the beating heart by utilizing the ICD lead as a fiducial marker. A precise definition of the treatment volume was achieved through a combination of EAM, cardiac MRI, and CT imaging. Given the uncertainty of heart motion during respiration, accurate tracking is essential to ensure treatment effectiveness and avoid damage to surrounding critical structures, such as the esophagus, lungs, and great vessels. This case is an example that such precision is both feasible and crucial for safe and effective cardiac SBRT, although longer follow-up and confirmation with more cases are warranted. Additionally, cardiac SBRT should be performed after thorough multidisciplinary discussion, involving collaboration among experts from radiation oncology, cardiology, and medical physics.

## Conclusions

Cardiac SBRT is an emerging treatment for refractory VT that combines advanced technologies from cardiology and radiation oncology, including EAM, the CK system with real-time motion tracking via Synchrony, and AI-driven cardiac reconstructions with technologies such as inHeart technology. When an ICD is present, it can serve as a fiducial marker, eliminating the need for invasive markers. Though case reports, series, and early-phase studies have demonstrated its feasibility, safety, and potential effectiveness, larger studies with extended follow-up are needed to confirm long-term outcomes and monitor for delayed toxicities such as pneumonitis and pericarditis. Our case report, which involved a six-month follow-up without adverse effects, underscores the importance of continued observation and highlights the value of a multidisciplinary approach involving radiation oncologists, cardiac electrophysiologists, and medical physicists for optimal patient care.
